# Effect of endogenous and exogenous EGF on the growth of EGF receptor-hyperproducing human squamous cell carcinoma implanted in nude mice.

**DOI:** 10.1038/bjc.1995.425

**Published:** 1995-10

**Authors:** Y. Kitagawa, M. Ueda, N. Ando, S. Ozawa, M. Kitajima

**Affiliations:** Department of Surgery, Keio University School of Medicine, Tokyo, Japan.

## Abstract

The effect of epidermal growth factor (EGF) on the biological behaviour of human tumours in vivo is still controversial. We investigated the effect of EGF on the growth of an EGF receptor-hyperproducing human epidermoid carcinoma, A431 tumour, and on a human small-cell lung carcinoma, H69 tumour, without detectable EGF receptor by using sialoadenectomised (sialex) mice as an endogenous EGF-suppressed animal model. The plasma EGF concentration in the sialex athymic mice was significantly lower than that in the sham-operated mice (P < 0.05). After exogenous EGF replacement with an implanted minipump, the plasma EGF concentration was significantly increased in both groups (P < 0.05). There was no significant difference between the body weight growth curves of sialex and sham-operated mice with and without EGF treatment. The tumour weight of A431, both estimated and measured in sialex mice, was significantly lower than that in sham-operated control mice (P < 0.05), and the growth of A431 tumour was significantly increased by exogenous EGF treatment (P < 0.05). Mitotic activity of these tumours detected by immunohistochemical staining for incorporated bromodeoxyuridine indicated a mitosis-stimulatory effect of endogenous and exogenous EGF on A431 tumours. In contrast to these findings on A431 tumours, a growth-stimulatory effect of endogenous and exogenous EGF was not observed in the H69 tumour. These results suggest a growth-promoting effect of physiological levels of endogenous EGF on EGF receptor-hyperproducing human tumours in vivo.


British Journal of Cancer (1995) 72, 865-868
© 1995 Stockton Press All rights reserved 0007-0920/95 $12.00

### Effect of endogenous and exogenous EGF on the growth of EGF receptor-hyperproducing human squamous cell carcinoma implanted in nude mice

Y Kitagawa, M Ueda, N Ando, S Ozawa and M Kitajima

*Department of Surgery, Keio University School of Medicine, 35 Shinanomachi Shinjuku-ku Tokyo 160, Japan.*

**Summary** The effect of epidermal growth factor (EGF) on the biological behaviour of human tumours *in vivo* is still controversial. We investigated the effect of EGF on the growth of an EGF receptor-hyperproducing human epidermoid carcinoma, A431 tumour, and on a human small-cell lung carcinoma, H69 tumour, without detectable EGF receptor by using sialoadenectomised (sialex) mice as an endogenous EGF-suppressed animal model. The plasma EGF concentration in the sialex athymic mice was significantly lower than that in the sham-operated mice ($P<0.05$). After exogenous EGF replacement with an implanted minipump, the plasma EGF concentration was significantly increased in both groups ($P<0.05$). There was no significant difference between the body weight growth curves of sialex and sham-operated mice with and without EGF treatment. The tumour weight of A431, both estimated and measured in sialex mice, was significantly lower than that in sham-operated control mice ($P<0.05$), and the growth of A431 tumour was significantly increased by exogenous EGF treatment ($P<0.05$). Mitotic activity of these tumours detected by immunohistochemical staining for incorporated bromodeoxyuridine indictated a mitosis-stimulatory effect of endogenous and exogenous EGF on A431 tumours. In contrast to these findings on A431 tumours, a growth-stimulatory effect of endogenous and exogenous EGF was not observed in the H69 tumour. These results suggest a growth-promoting effect of physiological levels of endogenous EGF on EGF receptor-hyperproducing human tumours *in vivo*.

**Keywords:** squamous cell carcinoma; epidermal growth factor; epidermal growth factor receptor; sialoadenectomy; bromodeoxyuridine labelling index

Epidermal growth factor (EGF) was originally isolated from a mouse submandibular gland (Carpenter and Cohen, 1979) and has various biological actions as a potent mitogen for the epidermis and several epithelial tissues (Hollenberg, 1979; Das, 1982; Fox *et al.*, 1982). Many transformed cells produce EGF-associated growth factors, such as transforming growth factor alpha (TGF-$\alpha$) which interacts with EGF receptors, and TGF-$\beta$ (Sporn and Roberts, 1984). Overexpression of EGF receptor has been shown to occur at a high incidence both in primary squamous cell carcinomas and in established cell lines such as A431 (Ozanne *et al.*, 1985; Gullick *et al.*, 1986; Ozawa *et al.*, 1987*a*). The v-*erb*B oncogene of avian erythroblastosis virus originates from part of the host cell EGF receptor gene (Ullrich *et al.*, 1984; Shimizu *et al.*, 1985). Thus, the involvement of the EGF and its receptors in transformation and progression of malignant cells has been suggested. Although there are several studies investigating the effect of EGF on the growth of EGF receptor-hyperproducing cell lines *in vitro* and *in vivo* (Gill and Lazer, 1981; Kamata *et al.*, 1986; Ozawa *et al.*, 1987*b*; Amagase *et al.*, 1990; Murayama, 1990), the physiological role of EGF on the EGF receptor-hyperproducing tumour growth remains obscure and still controversial. Tsutsumi *et al.* (1987*a*) demonstrated that EGF at the physiological level promotes the implantation and growth of spontaneous mouse mammary tumours in female nude mice. There have been no previous studies on the effect of changes in plasma EGF levels within the physiological range on the growth of human tumours which have various EGF receptor levels. The mouse submandibular gland is a rich source of EGF, and it is well established that sialoadenectomy lowers the circulating level of EGF in mice (Tsutsumi *et al.*, 1986, 1987*b*). In this study, we investigated the effect of endogenous EGF on EGF-hyperproducing human tumours using sialoadenectomised mice.

Correspondence: M Ueda
Received 27 February 1995; revised 18 May 1995; accepted 24 May 1995

### Materials and methods

### *Materials*

Mouse submandibular gland EGF was obtained from Toyobo (Osaka, Japan). Bromodeoxyuridine (BrdU) and anti-BrdU monoclonal antibodies were obtained from Ikeda (Osaka) and Becton Dickinson (Mountain View, CA, USA), respectively. Normal mouse IgG was obtained from Sigma (St Louis, MO, USA). Alzet mini-osmotic pumps were obtained from Alza (Palo Alto, CA, USA). $^{125}$I-labelled EGF (100 $\mu$Ci $\mu g^{-1}$) was purchased from Amersham International (Buckinghamshire, UK). All other reagents were of analytical grade.

### *Cells and cell culture*

The human epidermoid carcinoma cell line A431 was obtained from the Japan Cancer Research Resource Bank. The human small-cell lung carcinoma cell line H69 was kindly given to us by Dr S Hirohashi. The cell lines were maintained at 37°C in a humidified 5% carbon dioxide atmosphere in Dulbecco's modified Eagle medium (DMEM) supplemented with 10% fetal calf serum.

### *Animals and treatments*

Male athymic mice (BALB/c nu/nu) were obtained from Nisseiken, Oume (Tokyo, Japan), and were maintained in an aseptic environment. The submandibular glands were removed (or a sham operation was performed) from 5-week-old male nude mice under ether anaesthesia. Two weeks after the sialoadenectomy (sialex) or sham operation, tumour cells were subcutaneously (s.c.) inoculated. Cells (A431 or H69) in culture were detached by trypsinisation and washed with serum-free DMEM. Cell suspensions of $1 \times 10^8$ cells in 150 $\mu$l of phosphate-buffered saline (PBS) were injected bilaterally in the abdominal region of the athymic mice. On the next day Alzet mini-osmotic pumps containing 150 $\mu$g of mouse EGF dissolved in physiological saline, or 200 $\mu$l of physiological saline, were implanted s.c. into the backs of athymic mice. The rate of EGF administration was 7.5 ng of EGF $min^{-1}$. Body weight and tumour size were measured every day after tumour cell inoculation. The tumour weight was estimated by the following formula:

$$\text{Tumour weight} = \text{longer diameter} \times \text{shorter diameter}^2 \times 0.5$$

On the 10th day after the inoculation of tumour cells, the mice were sacrificed. Tumours were removed under sterile conditions, weighed and stored at −70°C until use. The mice were divided into four groups: sialex only, sialex and EGF replaced, sham operation only and sham-operation and EGF replaced. Seven mice (14 tumours) were used for each group.

*Radioimmunoassay of EGF*

Blood was collected in a heparinised syringe via the orbital artery under ether anaesthesia. The blood samples were chilled immediately to 4°C and centrifuged at 8700 *g* for 5 min, and the plasma was aspirated and stored at −70°C until assayed. Plasma EGF concentrations were determined by radioimmunoassay using $^{125}$I-labelled EGF (Amersham).

*BrdU staining*

The excised tumours were examined for DNA replicating cells by the avidin–biotin complex (ABC) method using anti-BrdU monoclonal antibody. BrdU (25 mg $kg^{-1}$) was injected into the peritoneal cavity of the mice 3 h before the tumour removal. The excised tumours were fixed in 70% ethanol, embedded in paraffin and cut into 4-$\mu$m sections. The sections were deparaffinised, immersed in 2 M hydrochloric acid for 30 min to denature the DNA, and neutralised by exposure to 0.1 M sodium borate for 10 min. The sections were then covered with 10% normal horse serum at room temperature for 30 min, and incubated with anti-BrdU monoclonal antibody (diluted 1:100 with 10% normal horse serum) at 4°C overnight. The subsequent steps were carried out according to the usual ABC staining method. The sections were stained with diaminobenzidine (0.5 mg $ml^{-1}$) in 0.01% hydrogen peroxide. Normal mouse IgG was used as a negative control. Labelling indices were obtained as follows: nuclei of 2000 tumour cells were classified as labelled or unlabelled. The labelling indices were then calculated from the equation:

$$\text{BrdU labelling index} = \frac{\text{Number of labelled tumour cell nuclei}}{\text{Number of tumour cell nuclei counted}} \times 100$$

*Statistical analysis*

Data were analysed by Student's *t*-test. A value of $P<0.05$ was considered significant.

### Results

*Effect of sialex on plasma EGF levels*

The plasma EGF concentration in the sialex mice (0.11 ± 0.03 ng $ml^{-1}$) was significantly lower than that in the sham-operated mice (2.15 ± 1.23 ng $ml^{-1}$) ($P<0.05$) (Figure 1). The plasma EGF levels in the sialex and sham-operated mice were significantly increased by continuous EGF administration. After the exogenous EGF replacement, there was no significant difference between the plasma EGF levels of the sialex (7.90 ± 1.81 ng $ml^{-1}$) and sham-operated mice (8.67 ± 1.54 ng $ml^{-1}$) (Figure 1).

*Effect of sialex and exogenous EGF replacement on tumour growth in athymic mice*

The growth curves for estimated tumour weight of A431 tumours in four different groups – sham-operated control, sham-operated control + EGF, sialex and sialex + EGF – are shown in Figure 2. Increase in tumour weight in the sialex mice was significantly suppressed compared with that in the sham-operated control mice ($P<0.05$). In both the EGF-treated sialex and EGF-treated sham-operated mice, growth of the tumours was significantly increased ($P<0.05$). The growth curves for H69 tumours in the four groups are shown in Figure 3. There was no significant difference in estimated tumour weight of these tumours among the four groups. These findings on estimated tumour growth in A431 and H69 tumours were confirmed by weighing the tumours after they were removed (Table I).

**Figure 1** Effect of sialoadenectomy and EGF replacement by minipump on plasma EGF levels. The plasma EGF concentrations (mean ± s.e.) of sham-operated mice (sham op.), sialoadenectomised mice (sialex), mice with sialoadenectomy and EGF replacement (sialex + EGF) and mice with sham operation and EGF replacement (sham op. + EGF) are shown. *$P<0.05$.

**Figure 2** Growth curves of the estimated tumour weight of the A431 tumour transplanted into nude mice. Growth curves of the estimated tumour weight of the A431 tumours transplanted into nude mice with sham operation (○), sialoadenectomy (●), sham operation and EGF replacement (△) and sialoadenectomy and EGF replacement (▲) are shown. *$P<0.05$.

*Effect of sialex and exogenous EGF replacement on mitotic activity of tumour cells detected by BrdU staining*

Tumour cells that had incorporated BrdU into DNA were detected by immunohistochemical staining using anti-BrdU monoclonal antibody. BrdU labelling indices of A431 and H69 tumours in these four groups are indicated in Table II. The BrdU labelling index of A431 tumours in the sialex mice was lower (not significantly) than that in the sham-operated control mice. BrdU labelling indices of EGF-treated sialex and sham-operated mice were significantly higher than that in the sham-operated control mice ($P<0.05$). On the other hand, there was no significant difference in the BrdU labelling indices of H69 tumours among the four groups, as shown in Table II.

### Discussion

Early studies on the effects of EGF on the growth of EGF receptor-hyperproducing squamous carcinoma cell lines in culture, including A431 cells, have demonstrated a growth-inhibitory effect of EGF on these cell lines (Gill and Laser, 1981; Kamata *et al*., 1986). Although KU1, a human bladder carcinoma cell line established from a high-grade transitional cell carcinoma, had an elevated number of EGF receptors compared with A431, anchorage-dependent growth of KU1 was not inhibited by EGF. Instead, anchorage-independent growth of KU1 was stimulated by EGF (Ishikawa *et al*., 1989). Previously, Lee *et al*. (1990) have demonstrated that EGF inhibits anchorage-dependent growth but stimulates anchorage-independent growth of human squamous carcinoma cell lines that overexpress EGF receptors. These studies suggest that the proliferative responses of EGF receptor-hyperproducing cells to EGF might be affected by other characteristics of cell lines and culture conditions. It seems that there are many factors which can affect the proliferative responses to EGF *in vivo*, such as interaction with interstitial tissues and other mediators. As Fidler (1990) demonstrated in studies on mechanisms of cancer metastasis, the outcome of metastasis depends on the interaction of metastatic cells with different organ environments. Accordingly, from this point of view, studies of 'the *in vivo* environment' are important for the investigation of actual phenomena in human carcinogenesis and progression.

However, the effect of EGF on the proliferation of EGF receptor-hyperproducing tumours *in vivo* is still complicated and controversial. Murayama (1990) has reported the growth-inhibitory effects of EGF on human breast cancer and oesophageal cancer cells transplanted into athymic mice, and proposes the efficacy of EGF therapy for human cancers with overexpression of EGF receptor. Amagase *et al*. (1990) observed prolongation of the survival time of mice bearing various murine syngeneic tumours as well as athymic mice bearing human xenografts. On the other hand, Ginsburg and Vonderhaar, (1985) and Ozawa *et al*. (1989) demonstrated that EGF promotes the growth of tumours of EGF receptor-hyperproducing squamous carcinoma cells.

The studies cited above indicate that differences in the responses of EGF receptor-hyperproducing tumours to exogenous EGF might depend on the doses and routes of EGF administration. In this study, we have demonstrated the proliferative effects of endogenous EGF at a physiological level on the EGF receptor-hyperproducing human squamous carcinoma cells transplanted to athymic mice. The growth-inhibitory effect of sialex and growth-stimulatory effect of exogenous continuous EGF administration on EGF receptor-hyperproducing A431 tumours and the lack of an effect on EGF receptor-undetectable H69 tumours clearly imply a role of the EGF-EGF receptor system in the proliferation of EGF receptor-overexpressing human tumours. On the other hand, when EGF was administered as a local bolus injection directly into the tumour (Murayama, 1990), this induced down-regulation of the EGF receptor and did not reflect the physiological role of endogenous EGF. Also, Amagase *et al*. (1990) noted that prolongation of survival times by EGF is independent of the number of EGF receptors on tumour cells. The growth-inhibitory effects observed in these studies apparently depend on mechanisms other than the EGF-EGF receptor system. Tsutsumi *et al*. (1987*a*), however, demonstrated the promoting effect of a physiological level of EGF on the implantation and growth of a mouse mammary tumour in sialex nude mice. This is in agreement with our data, which are the first to demonstrate the growth-promoting effect of endogenous EGF on a human tumour using a sialex nude mice model.

In this study, the BrdU labelling indices in these tumours suggest that EGF promotes the mitotic activity of EGF receptor-hyperproducing tumours. Changes in tumour weight and BrdU labelling indices corresponded to the plasma EGF level only in the EGF receptor-hyperproducing A431 cell tumour. It is conceivable that physiological levels of EGF contribute to the activation of the signal transduction pathway that initiates cell proliferation.

Previously, squamous carcinomas of the neck, lung, gingiva and oesophagus were found to express elevated levels of EGF receptors at high frequency (Ozanne *et al*., 1985; Gullick *et al*., 1986; Ozawa *et al*., 1987*a*). Notably, in oesophageal squamous cell carcinoma, a significant correlation between the EGF receptor overexpression and poor prognosis of the disease has been reported (Ozawa *et al*., 1989). Amplification of the EGF receptor gene, c-*erb*B, was significantly correlated with lymph node metastasis and

**Figure 3** Growth curves of the estimated tumour weight of the H69 tumour transplanted into nude mice. Growth curves of the estimated tumour weight of the H69 tumours transplanted into nude mice with sham operation (○), sialoadenectomy (●), sham operation and EGF replacement (△) and sialoadenectomy and EGF replacement (▲) are shown.

**Table I** Tumour weight (g) at sacrifice of the four groups on the 10th day after subcutaneous inoculation of tumour cells

| | *Sialex* | *Sialex + EGF* | *Sham op.* | *Sham op. + EGF* |
|---|---|---|---|---|
| | ┌── * ──┐ | | ┌── * ──┐ | |
| A431 | 1.76 ± 0.23 | 4.35 ± 0.43 | 2.95 ± 0.33 | 4.41 ± 0.47 |
| H69 | 0.19 ± 0.19 | 0.22 ± 0.23 | 0.17 ± 0.36 | 0.20 ± 0.42 |

*$P<0.05$.

**Table II** BrdU labelling indices[a] in tumours implanted into athymic mice

| | *Sialex* | *Sialex + EGF* | *Sham op.* | *Sham op. + EGF* |
|---|---|---|---|---|
| | ┌── ** ──┐ | | ┌── ** ──┐ | |
| A431 | 4.2 ± 2.5 | 13.1 ± 3.2 | 5.6 ± 2.1 | 15.5 ± 3.1 |
| H69 | 2.0 ± 1.9 | 1.9 ± 1.5 | 1.5 ± 0.9 | 2.2 ± 1.7 |

[a]BrdU labelling index = (Number of labelled tumour cell nuclei/Number of tumour cell nuclei counted) × 100. **$P<0.05$.

poorer prognosis in patients with oesophageal squamous cell carcinoma (Kitagawa *et al.*, 1992). Moreover, EGF expression has been detected in several human cancer cells, including squamous cell carcinoma. Our observations in this study suggest that EGF receptor-hyperproducing carcinomas in patients have growth advantages through the EGF–EGF receptor system, including the autocrine loop.

On the basis of such findings, Hirota *et al.* (1989) have demonstrated the inhibitory effect of an immunotoxin, which is the conjugated form of anti-EGF receptor monoclonal antibody and plant toxin, to the A431 tumours transplanted in athymic mice as a model of the anti-EGF–EGF receptor system treatment. The findings shown in this study also are encouraging for the establishment of anti-EGF–EGF receptor system treatments for EGF receptor-hyperproducing human cancers.

### References

AMAGASE H, TAMURA K, OKUHIRA M, KAKIMOTO M, AMANO H, HASHIMOTO K, FUWA T AND TSUKAGOSHI S. (1990). Epidermal growth factor prolongs survival time of tumor-bearing mice. *Jpn J. Cancer Res.*, **81,** 495–500.

CARPENTER G AND COHEN S. (1979). Epidermal growth factor. *Annu. Rev. Biochem.*, **48,** 193–216.

DAS M. (1982). Epidermal growth factor: mechanism of action. (1982). *Int. Rev. Cytol.*, **78,** 233–256.

FIDLER IJ. (1990). Critical factors in the biology of human cancer metastasis. *Cancer Res.*, **50,** 6130–6138.

FOX CF, LINSEY PS AND WRANN M. (1982). Receptor remodeling and regulation in the action of epidermal growth factor. *Fed. Proc.*, **41,** 2988–2995.

GILL GN AND LAZER CS. (1981). Increased phosphotyrosine content and inhibition of proliferation in EGF treated A431 cells. *Nature*, **293,** 305–307.

GINSBURG E AND VONDERHAAR BK. (1985). Epidermal growth factor stimulates the growth of A431 tumors in athymic mice. *Cancer Lett.*, **28,** 143–150.

GULLICK WJ, MARSDEN JJ, WITTLE N, WARD B, BOBROW B AND WATERFIELD MD. (1986). Expression of epidermal growth factor receptors on human cervical, ovarian, and vulval carcinomas. *Cancer Res.*, **46,** 285–292.

HIROTA N, UEDA M, OZAWA S, ABE O AND SHIMIZU N. (1989). Suppression of an epidermal growth factor receptor-hyperproducing tumor by an immunotoxin conjugate of gelonin and a monoclonal anti-epidermal growth factor receptor antibody. *Cancer Res.*, **49,** 7106–7109.

HOLLENBERG MD. (1979). Epidermal growth factor–urogastrone, a polypeptide aquiring hormonal status. *Vitam. Horm.*, **37,** 69–110.

ISHIKAWA J, MAEDA S, SUGIYAMA T, NISHIMURA R, MIZOGUCHI A AND KAMIDONO S. (1989). EGF stimulates anchorage-independent growth of a receptor gene. *Int. J. Cancer*, **44,** 1000–1004.

KAMATA N, CHIDA K, RIKIMARU K, HORIKOSHI M, ENOMOTO S AND KUROKI T. (1986). Growth-inhibitory effect of epidermal growth factor and overexpression of its receptors on human squamous cell carcinomas in culture. *Cancer Res.*, **46,** 1648–1653.

KITAGAWA Y, UEDA M, ANDO N, OZAWA S AND KITAJIMA M. (1992). Prognostic significance of EGF receptor gene amplification in patients with esophageal squamous carcinoma. *Eur. Surg. Res.*, **24,** 101–102.

LEE K, TANAKA M, SHIGENO C, YAMAMOTO I, OHTA S, RIKIMARU K, HATANAKA M AND KONISHI J. (1990). Epidermal growth factor stimulates the anchorage-independent growth of human squamous cell carcinomas overexpressing its receptors. *Biochem. Biophys. Res. Commun.*, **168,** 905–911.

MURAYAMA Y. (1990). Growth-inhibitory effects of epidermal growth factors on human breast cancer and carcinoma of the esophagus transplanted into nude mice. *Ann. Surg.*, **211,** 263–268.

OZANNE B, SHUM A, RICHARDS CS, CASSELS D, GROSSMANN D, TRENT J, GUSTERSON B AND HENDLER F. (1985). Evidence for an increase of EGF receptors in epidermoid malignancies. *Cancer Cells*, **3,** 41–49.

OZAWA S, UEDA M, ANDO N, ABE O AND SHIMIZU N. (1987*a*). High incidence of EGF hyperproduction in oesphageal squamous cell carcinomas. *Int. J. Cancer*, **39,** 333–337.

OZAWA S, UEDA M, ANDO N, ABE O, HIRAI M AND SHIMIZU N. (1987*b*). Stimulation by EGF of the growth of EGF receptor-hyperproducing tumor cells in athymic mice. *Int. J. Cancer*, **40,** 706–710.

OZAWA S, UEDA M, SHIMIZU N AND ABE O. (1989). Prognostic significance of epidermal growth factor receptor in esophageal squamous cell carcinomas. *Cancer*, **63,** 2169–2173.

SPORN MB AND ROBERTS AB. (1984). Autocrine growth factors and cancer. *Nature*, **313,** 745–747.

SHIMIZU N, HUNTS J, MERLINO G, WANG-PENG J, XU Y-H, YAMAMOTO T, TOYOSHIMA K AND PASTAN I. (1985). Regional mapping of the EGF receptor(EGFR)/c-*erb*-B protooncogene. *Cytogenet. Cell Genet.*, **40,** 743–744.

TSUTSUMI O, TSUTSUMI A AND OKA T. (1987*a*). Importance of epidermal growth factor in implantation and growth of mouse mammary tumor in female nude mice. *Cancer Res.*, **47,** 4651–4653.

TSUTSUMI O, KUBOTA Y AND OKA T. (1987*b*). Effect of sialoadenectomy, treatment with epidermal growth factor (EGF) antiserum and replacement of EGF on the epidermis in mice. *J. Endocrinol*, **113,** 193–197.

TSUTSUMI O, KURACHI H AND OKA T. (1986). A physiological role of epidermal growth factor in male reproductive function. *Science*, **233,** 975–977.

ULLRICH A, COUSSENS L, HAYFLICK JS, DULL TJ, GRAY A, TAM AW, LEE J, YARDEN Y, LIBERMANN TA, SCHLESSINGER J, DOWNWARD J, MAYES EL, WHITTLE N, WATERFIELD MD AND SEEBURG PH. (1984). Human epidermal growth factor receptor cDNA sequence and aberrant expression of the amplified gene in A431 epidermoid carcinoma cells. *Nature*, **309,** 418–425.


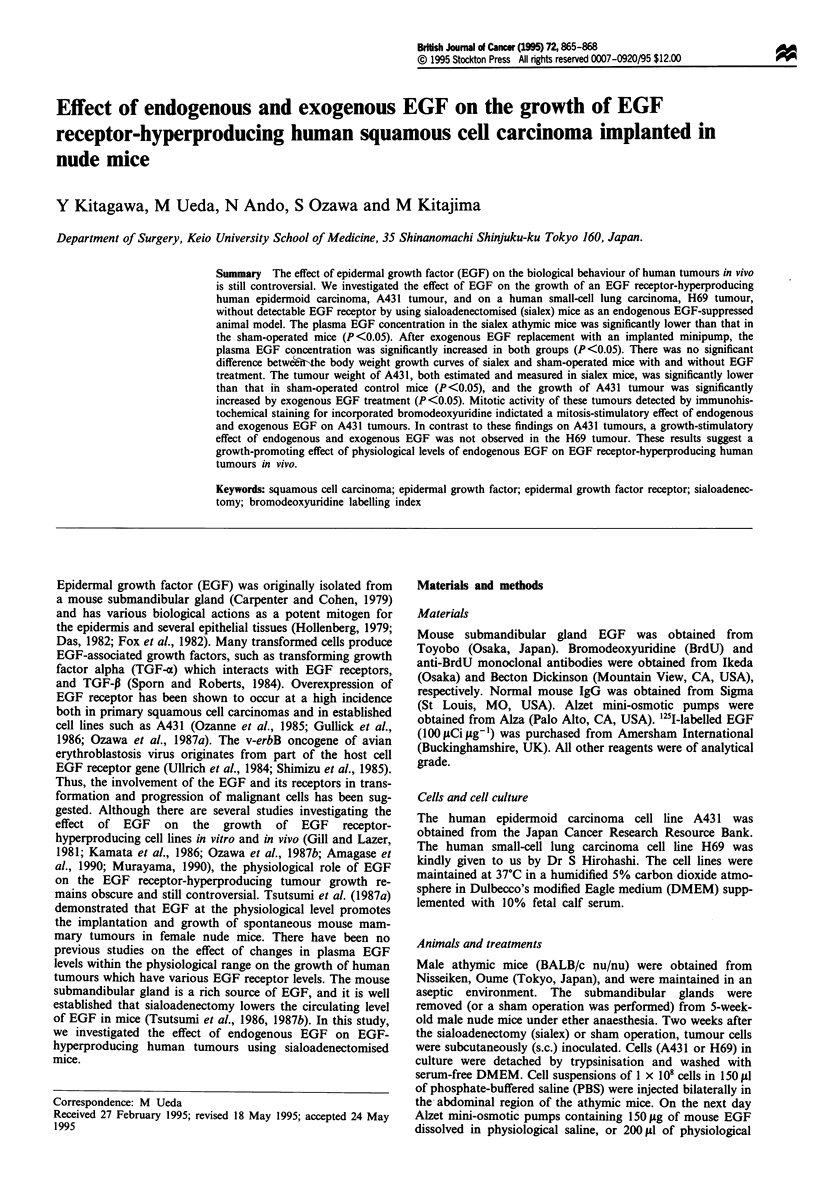

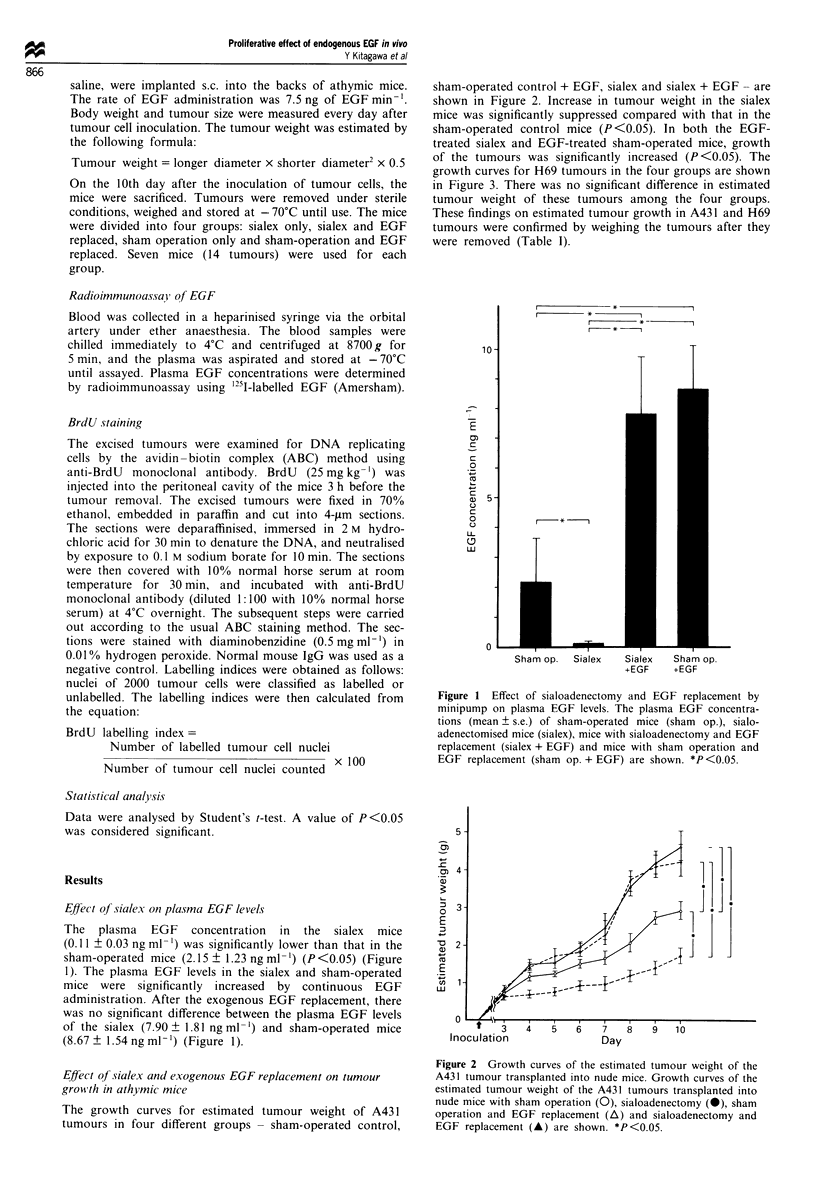

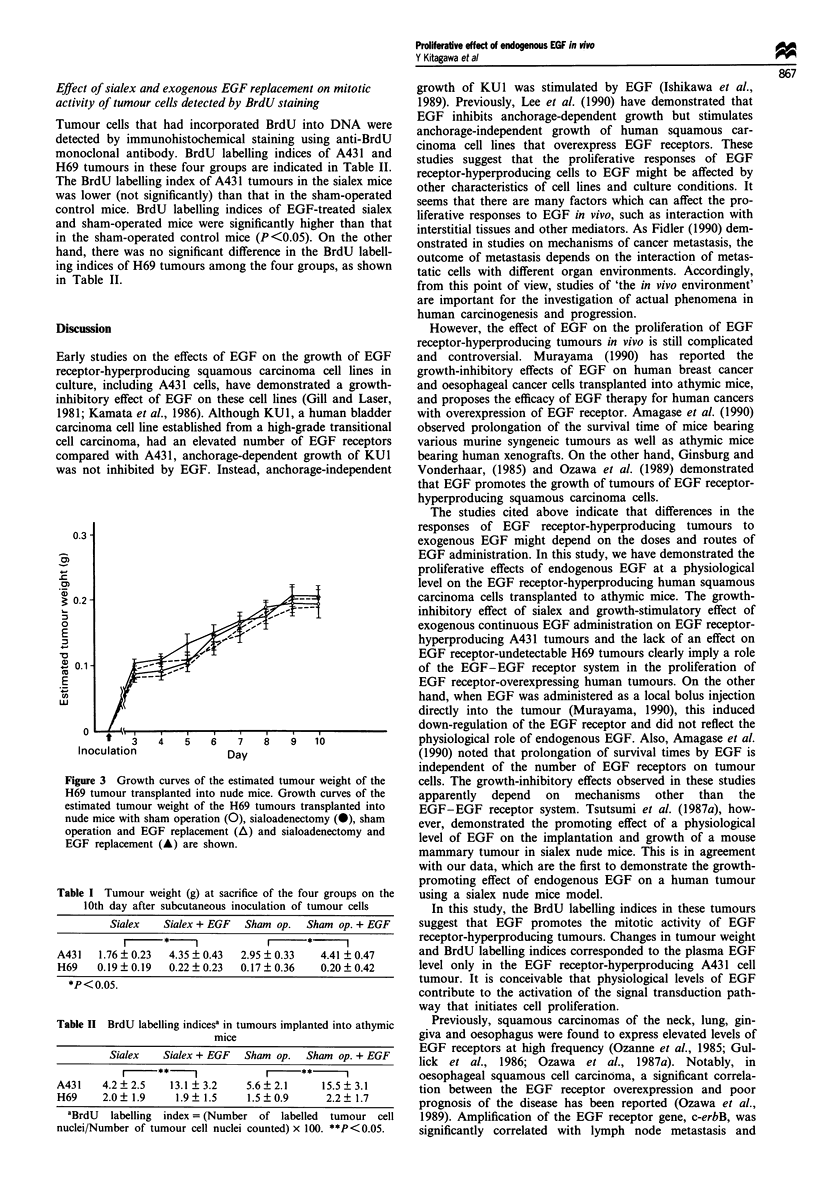

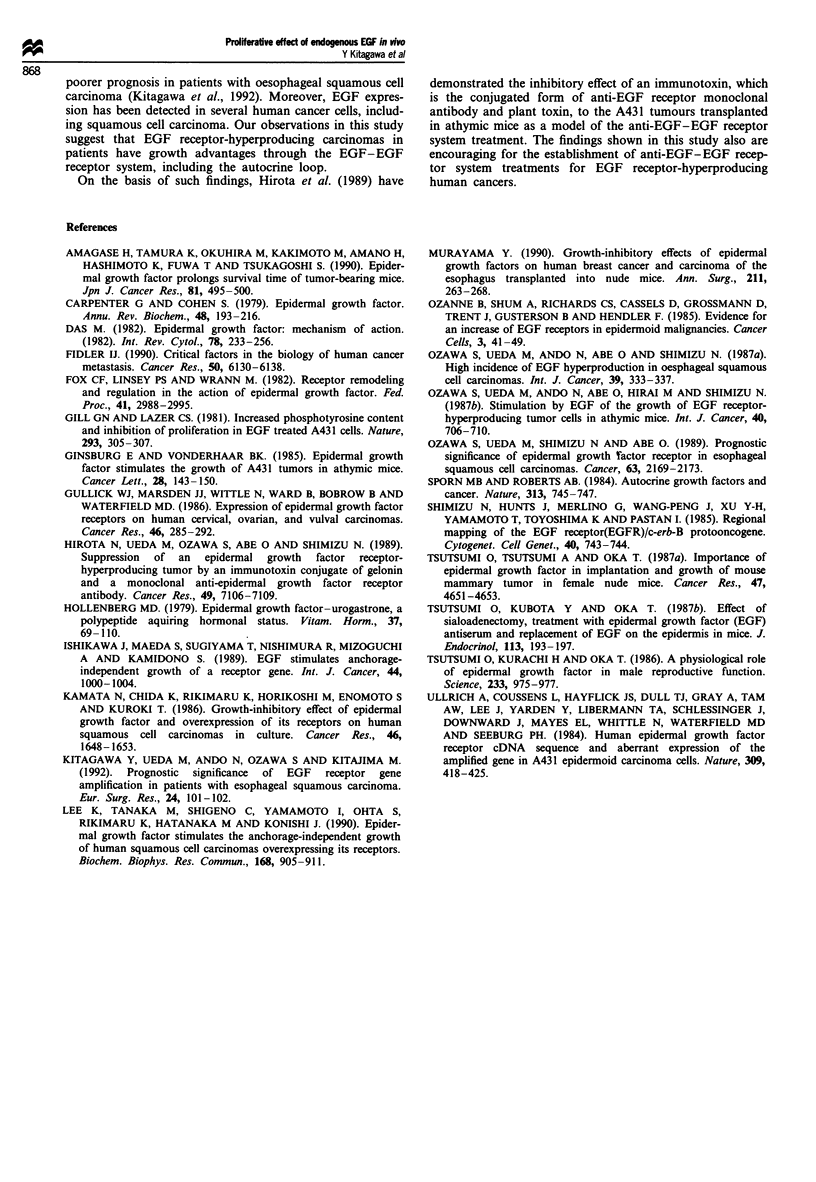


## References

[OCR_00499] Amagase H., Tamura K., Okuhira M., Kakimoto M., Amano H., Hashimoto K., Fuwa T., Tsukagoshi S. (1990). Epidermal growth factor prolongs survival time of tumor-bearing mice.. Jpn J Cancer Res.

[OCR_00507] Carpenter G., Cohen S. (1979). Epidermal growth factor.. Annu Rev Biochem.

[OCR_00511] Das M. (1982). Epidermal growth factor: mechanisms of action.. Int Rev Cytol.

[OCR_00513] Fidler I. J. (1990). Critical factors in the biology of human cancer metastasis: twenty-eighth G.H.A. Clowes memorial award lecture.. Cancer Res.

[OCR_00519] Fox C. F., Linsley P. S., Wrann M. (1982). Receptor remodeling and regulation in the action of epidermal growth factor.. Fed Proc.

[OCR_00522] Gill G. N., Lazar C. S. (1981). Increased phosphotyrosine content and inhibition of proliferation in EGF-treated A431 cells.. Nature.

[OCR_00529] Ginsburg E., Vonderhaar B. K. (1985). Epidermal growth factor stimulates the growth of A431 tumors in athymic mice.. Cancer Lett.

[OCR_00534] Gullick W. J., Marsden J. J., Whittle N., Ward B., Bobrow L., Waterfield M. D. (1986). Expression of epidermal growth factor receptors on human cervical, ovarian, and vulval carcinomas.. Cancer Res.

[OCR_00540] Hirota N., Ueda M., Ozawa S., Abe O., Shimizu N. (1989). Suppression of an epidermal growth factor receptor-hyperproducing tumor by an immunotoxin conjugate of gelonin and a monoclonal anti-epidermal growth factor receptor antibody.. Cancer Res.

[OCR_00547] Hollenberg M. D. (1979). Epidermal growth factor-urogastrone, a polypeptide acquiring hormonal status.. Vitam Horm.

[OCR_00553] Ishikawa J., Maeda S., Sugiyama T., Nishimura R., Mizoguchi A., Kamidono S. (1989). EGF stimulates anchorage-independent growth of a human bladder carcinoma cell line (KU1) with an amplified and over-expressed EGF receptor gene.. Int J Cancer.

[OCR_00559] Kamata N., Chida K., Rikimaru K., Horikoshi M., Enomoto S., Kuroki T. (1986). Growth-inhibitory effects of epidermal growth factor and overexpression of its receptors on human squamous cell carcinomas in culture.. Cancer Res.

[OCR_00572] Lee K., Tanaka M., Shigeno C., Yamamoto I., Ohta S., Rikimaru K., Hatanaka M., Konishi J. (1990). Epidermal growth factor stimulates the anchorage-independent growth of human squamous cell carcinomas overexpressing its receptors.. Biochem Biophys Res Commun.

[OCR_00578] Murayama Y. (1990). Growth-inhibitory effects of epidermal growth factor on human breast cancer and carcinoma of the esophagus transplanted into nude mice.. Ann Surg.

[OCR_00595] Ozawa S., Ueda M., Ando N., Abe O., Hirai M., Shimizu N. (1987). Stimulation by EGF of the growth of EGF receptor-hyperproducing tumor cells in athymic mice.. Int J Cancer.

[OCR_00588] Ozawa S., Ueda M., Ando N., Abe O., Shimizu N. (1987). High incidence of EGF receptor hyperproduction in esophageal squamous-cell carcinomas.. Int J Cancer.

[OCR_00601] Ozawa S., Ueda M., Ando N., Shimizu N., Abe O. (1989). Prognostic significance of epidermal growth factor receptor in esophageal squamous cell carcinomas.. Cancer.

[OCR_00604] Sporn M. B., Roberts A. B. Autocrine growth factors and cancer.. Nature.

[OCR_00622] Tsutsumi O., Kubota Y., Oka T. (1987). Effect of sialoadenectomy, treatment with epidermal growth factor (EGF) antiserum and replacement of EGF on the epidermis in mice.. J Endocrinol.

[OCR_00626] Tsutsumi O., Kurachi H., Oka T. (1986). A physiological role of epidermal growth factor in male reproductive function.. Science.

[OCR_00616] Tsutsumi O., Tsutsumi A., Oka T. (1987). Importance of epidermal growth factor in implantation and growth of mouse mammary tumor in female nude mice.. Cancer Res.

[OCR_00631] Ullrich A., Coussens L., Hayflick J. S., Dull T. J., Gray A., Tam A. W., Lee J., Yarden Y., Libermann T. A., Schlessinger J. Human epidermal growth factor receptor cDNA sequence and aberrant expression of the amplified gene in A431 epidermoid carcinoma cells.. Nature.

